# The Development of a Surface Finisher of Car Park Slab Using Waterborne Silicon Acrylic with Polyamide [Part II: Safety Tests]

**DOI:** 10.3390/ma12010158

**Published:** 2019-01-06

**Authors:** Junho Gong, Hoseong Jeong, Dooyong Cho

**Affiliations:** Department of Convergence System Engineering, Chungnam National University, Daejeon 34134, Korea; jhgong@cnu.ac.kr (J.G.); hsjeong@cnu.ac.kr (H.J.)

**Keywords:** surface finisher, car park, waterborne silicon acrylic, polyamide, safety test, eco-friendly

## Abstract

Due to the environmental concerns of solventborne coating systems, environmental directives have recently been promulgated in many countries. Additionally, integrated environmental policies have been pushed in many fields to minimise influences on the environment. Waterborne silicon acrylic finishers have gained much interest to replace the traditional finishing system. To satisfy the requirements, a waterborne finisher with polyamide was previously developed and its performance was determined. For further safety assessment, various tests were conducted, such as gas toxicity, heavy metals tests, chemical resistance test and chloride migration test, followed by equivalent standards. In the cases of gas toxicity and heavy metals evaluations, both results were acceptable considering their corresponding standards, e.g. KS F 2271, KS F 3888-2 and BS EN 71-3. Based on the evaluation, silicon acrylic with 30% mix ratio of polyamide resin (SA+PR30%) could be implemented as an environmentally friendly finisher for various applications. In the chemical resistance and chloride migration test results, the developed finisher showed a barrier effect in the chemical environment. Thus, the developed finisher could be an alternative finisher applicable for slabs in chemical industrial areas.

## 1. Introduction

Surface finishing materials are extensively used in various fields of applications, especially car park slabs, because underground car park concrete pavement has the major environmental issue of fine dust creation by repetitive loads from tires and the lack of air circulation system. Among the current techniques to prevent this issue, the implementation of organic coatings is considered as an economical approach to offer a comfortable underground car park condition to customers [1,2,3].

Typical top finishers such as epoxy and urethane, which are solventborne organic, are well-known and commonly applied in practice because they are cost effective, durable, waterproof, and non-slip [2,4]. However, environmental issues, particularly the transmission of Volatile Organic Compounds (VOCs) to the air, are associated with the application [5]. For instance, urethane and epoxy coatings can lead to health and safety issues because a volatile organic solvent is mixed with urethane or epoxy finishers to increase workability when under construction. Uncomfortable scent and environmental hormones from the solvent or epoxy, in an enclosed working environment, mean workers could be subjected to dangerous situations and fire incidents might occur [1,2].

Due to these concerns, many countries have promulgated environmental directives to decrease airborne pollution from VOCs [6,7]. Among the alternatives of solvent based surface finishers, waterborne finishing materials have emerged as an eco-friendly substitution [5,8,9]. In the early stage of research on waterborne coatings, poor hardness properties of waterborne finishers was one of the critical defects compared to traditional finishers as well as the trade-off between lower VOCs and better structural performance, which is an aim of waterborne finisher. To deal with this problem, a study on hybrid technology of acrylic and urethane was conducted [10,11,12].

The objective of this study was to evaluate chemical safety of the previously developed and evaluated surface finisher for car parks with a hybrid technology of silicon acrylic and polyamide resin to decrease VOCs emission and enhanced properties. In the preceding study, mechanical and chemical properties of the developed finisher were evaluated followed by KS (Korean Standard) F 4937. It was found that the finisher passed the evaluation criteria of KS F 4937 including adhesion by pull-off test, impact resistance test, permeability test, abrasive wear resistance to wheel-moving test, and pollutant emission test. For the safety test, diverse evaluation criteria such as gas toxicity, heavy metal, chemical resistance, and chloride migration were adopted to determine and provide baseline data for development of eco-friendly top finisher [13].

## 2. Materials and Mix Design

### 2.1. Waterborne Silicon Acrylic Emulsion

A waterborne surface finisher developed by a Chinese company was introduced by Eco Sports Chemical Technology Company (GyeongGi-do, Korea). The chemical composition of the finisher is as follows: 46–48% of silicon acrylic (SA) resin, 52–54% of water and 1–2% of polyoxyethylene pentylphenol ether. This finisher is currently used for coating slabs for sports fields.

### 2.2. Polyamide Resin

The polyamide resin (PR) provided by Jevisco Company (Seoul, Korea) was previously adopted as a resin to develop a hybrid technology with waterborne silicon acrylic emulsion to enhance the performance of the waterborne surface finisher for car park slabs. This material, a mixture of modified polyamide and hardener with ratio of 4:1, is broadly used as a primer for slab sealing in architectural and civil engineering industries. The polyamides have diverse characteristics such as high strength, abrasion resistance, resilience, and good hydrophilicity. Due to these characteristics, polyamides are often implemented in the manufacture of clothing and carpets. Additionally, they are combined with fillers, pigments, glass fiber and toughening agents to enhance mechanical strength and barrier properties to the polymer [14].

### 2.3. Mix Design

The performance of developed surface finisher was previously determined by evaluation criteria such as adhesion strength, impact resistance, permeability, and pollutant emission from KS F 4937: Korean standard for surface finishing material for car park slab. The silicon acrylic emulsion with mix ratio 30% of polyamide resin (SA+PR30%) (Table 1) satisfied all evaluation criteria in KS F 4937 and is considered a candidate for safety tests.

## 3. Experimental Programme

### 3.1. Testing Method for Gas Toxicity of Finish Materials of Buildings (KS F 2271)

**Figure 1:**
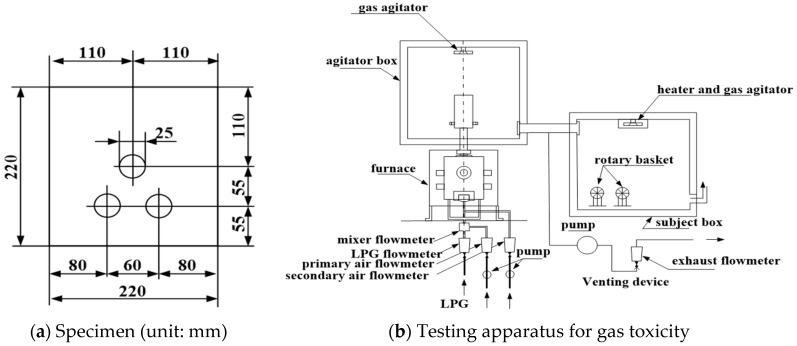
Configuration of testing specimen and apparatus for gas toxicity.

The SA+PR30% was spread out onto the surface of two fiber-reinforced calcium silicate boards and three holes that penetrate from the front surface to the rear surface of each specimen were made, as shown in Figure 1a. Preliminary heating of the furnace was applied by burning a fiber-reinforced calcium silicate board with air flow rate of 3.0 L/min and 25.0 L/min through the primary and secondary supply devices, respectively, as illustrated in Figure 1b. The subject box was filled with the burning gas and heating test commenced as temperature of the box dropped to 30 °C. Then, eight rotary baskets with eight white lab mice were prepared to put in the subject box. The mean elapsed time was measured from the commencement of heating to when the lab mice stopped moving. The tests were conducted twice and lasted 15 min each.

### 3.2. Heavy Metals Test

The KS F 3888-2 is a quality standard to determine physical properties, heavy metals, and composition of harmful substances of paving materials for diverse outdoor facilities such as in sports facilities and public areas. This standard includes the four crucial heavy metals Pb, Cd, Cr^+6^, and Hg, 15 other heavy metals, and six types of phthalate plasticisers referred in BS EN 71-3: Safety of toys. Migration of certain elements is currently considered as evaluation criteria. To justify compatibility of the developed surface finisher, the four main heavy metals, PAHs, the 15 other heavy metals, and the phthalate plasticisers were examined by implementing the methods of KS M 6556, BS EN 71-3, and KS M 1991, respectively. The limitation of each evaluation criteria is indicated in Table 2.

### 3.3. Chemical Resistance Test

**Figure 2:**
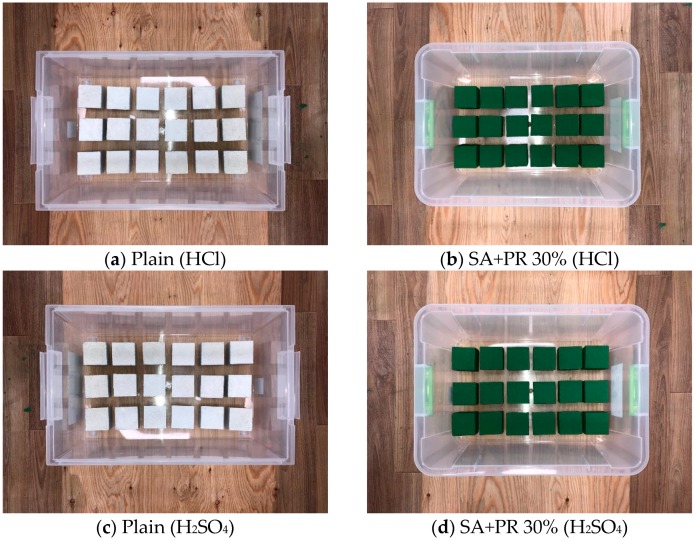
Arrangement of specimens for chemical resistance test.

The relative chemical resistance of the developed surface finisher was verified in accordance with ASTM-C267: Standard test method for chemical resistance of mortars. The surface of the mortar with dimensions of 50 m × 50 m × 50 mm was painted with the finisher and cured at room temperature. The chemical environmental conditions were the following: 5% sulphuric acid (H_2_SO_4_) and 5% hydrochloric acid (HCl) respectively. The plain and SA+PR30% specimens were submerged into the chemical conditions for 28 days, as shown in Figure 2. After 1, 7, 14, and 28 days, the immersed specimens were cleaned with running water and dehydrated at room temperature. The chemical resistance was evaluated by measuring the weight loss of the specimens.

### 3.4. Chloride Migration Test

**Figure 3:**
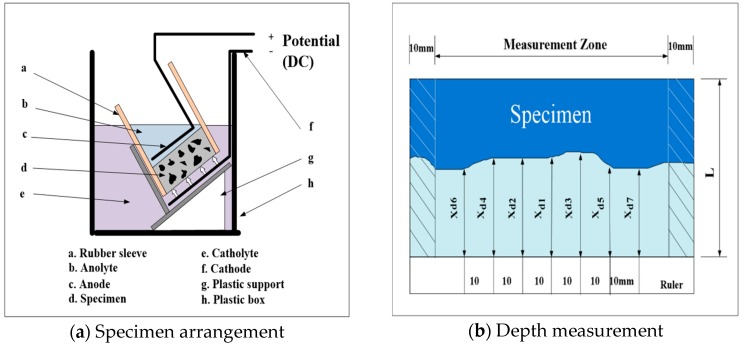
Schematic arrangement of the migration test.

To validate the resistance performance of chloride migration of the top finisher, a non-steady-state migration test was conducted as described in NT Build 492. Concrete cylinders with a diameter of 100 mm and a height of 50 mm were prepared and top coated by the developed finisher. Then, the cylinders were submerged into the container filled with calcium hydroxide (Ca(OH)_2_) solution for 18 h. Once the specimens were arranged for the test, they were fit by rubber sleeve and immerged in the catholyte reservoir filled with a 10% Sodium chloride (NaCl) solution, as indicated in Figure 3a. Afterwards, the anolyte solution, which was sodium hydroxide (NaOH), was poured in the sleeve above the specimens. The tests progressed with applied voltage of 20 V for 24 h. The cylinders were split into two pieces and silver nitrate (AgNO_3_) solution was sprayed onto the split profile. As the white silver chloride precipitation is clearly visible, the depth of chloride penetration was measured from the centre to both edges at intervals of 10 mm, as illustrated in Figure 3b.

## 4. Results

### 4.1. Testing Method for Gas Toxicity of Finish Materials of Buildings

The gas toxicity test was conducted to determine the gas toxicity of the developed finisher by exposing lab mice to the combustion gas according to KS F 2271. The stopping times of lab mice were recorded from the commencement of the test. Table 3 indicates the gas toxicity test result. The average stopping times of the tests were 14 min 45 s and 14 min 41 s, respectively. The elapsed times exceeded the standard of KS F 2271, which is 9 min.

### 4.2. Heavy Metals Test

The heavy metals test was performed to detect the amount of Polycyclic Aromatic Hydrocarbon (PAHs), heavy metals, and phthalate plasticisers within the developed finisher. The previously developed surface finisher was examined followed by tests according to KS M 6956, BS EN 71-3 and KS M 1991. As listed in Table 4, none of the PAHs, heavy metals or phthalate plasticisers were detected in the tests, except 10 mg/kg of lead, which is far below the standard (90 mg/kg).

### 4.3. Chemical Resistance Test

**Figure 4:**
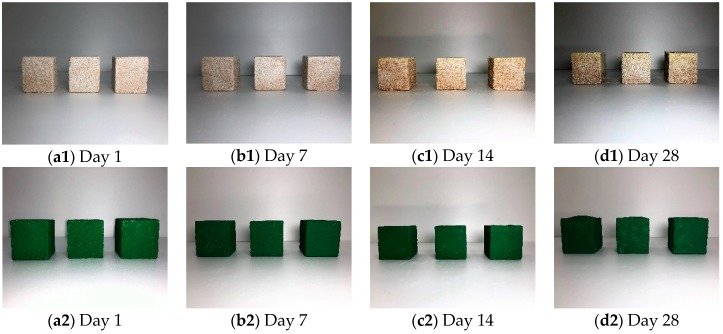
Specimens submerged in hydrochloric acid.

**Figure 5:**
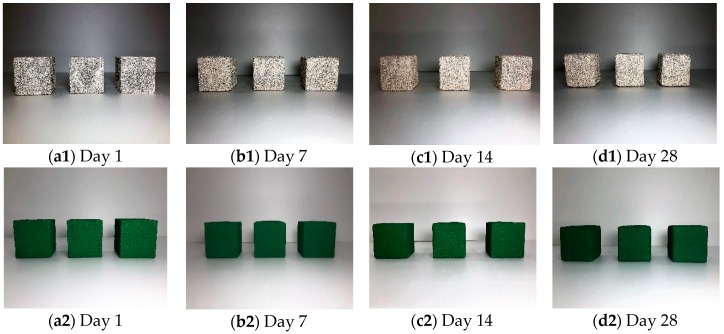
Specimens submerged in sulphuric acid.

**Figure 6:**
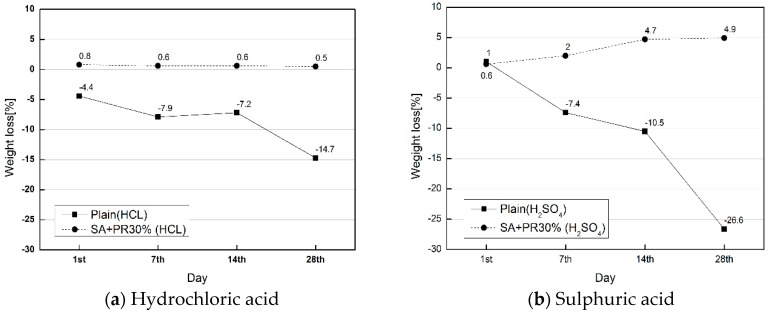
Weight loss of specimens.

The chemical resistance test was designed to evaluate chemical resistance of SA+PR30% followed by ASTM C 267. The plain and coated cubic mortar specimens in chemical environments of HCl and H_2_SO_4_ were dried out on Days 1, 7, 14, and 28 from the commencement of chemical resistance test to observe surface appearance and to measure weight loss of specimens. Figure 4 and Figure 5 illustrate the appearance of the specimens in HCl and H_2_SO_4_, respectively. The plain specimens aggressively reacted with both HCl and H_2_SO_4_ and a deterioration of fine aggregate occurred at the surface after seven days. The SA+PR30% specimens in the both chemical environments showed the internal expansion of the finisher after 28 days. It was determined that the expansion developed by the chemical reaction between mortar and chemical solutions penetrating through the microscopic gaps at the edges of specimens. Figure 6 shows the average weight loss of both plain and finished specimens submerged in chemical conditions. In the case of SA+PR30%, unless they were internally expanded due to medium penetration, the weight of specimens slightly increased. However, there were dramatic weight losses in the plain specimens in both chemical environments, with 15% weight loss in sulphuric acid between Days 14 and 28.

### 4.4. Chloride Migration Test

**Figure 7:**
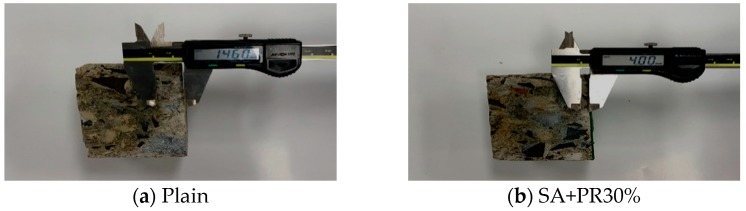
Chloride penetration measurement.

To evaluate resistance of chloride ion, chloride migration test according to NT Build 492 was performed by measuring and calculating the migration and its coefficient, respectively. Figure 7 indicates the thickness of penetration measured in the centre of both plain and SA+PR 30%. From the depth measurement, the difference of average migrated thickness between the two types of specimens was around 11.5 mm, approximately 78% of the plain specimen. Additionally, non-steady-state migration coefficients of both plain and SA+PR30% were simply calculated by substituting the factors in Table 5 into Equation (1) from NT Build 492 to compare and evaluate chloride resistance of the developed finisher. Throughout the migration coefficient calculation, the penetration coefficient from SA+PR 30% was retarded up to roughly 18% compared to plain specimen.

(1) $$D_{\mathrm{nssm}}=\frac{0.0239(273+7)L}{(U-2)t}\left(x_{d}-0.0238\sqrt{\frac{(273+T)Lx_{d}}{U-2}}\right)$$

## 5. Discussion

All outcomes of safety tests conducted in this study showed that SA+PR30% is eco-friendly and has chemical and chloride resistance properties. In the previous study for developing and evaluating the surface finisher, it was determined that prolongation of service life of the waterborne finisher was accomplished by introducing PR. Due to aforementioned properties of the polyamide, adhesion strength, for instance, was increased depending on the amount of PR. This development of mechanical property could be attributed to the barrier effect by retarding chemical and chloride penetration to the substrate.

Although the developed finisher affected the chemical resistance, there were slight internal expansions of specimens submerged in H_2_SO_4_ and weight change occurred. This would be expected as there were microscopic gaps at the edges of specimens, which were made during the preparation of specimens. Additionally, the increased weight might be influenced by a continuous formation of Ettringite, which reacted with SO_4_^2−^ ion and monosulphate. Although there was no Pb content within the SA+PR30%, it was detected in the heavy metal test result. The Pb might have been introduced from SiO_2_ into SA+PR30%. Additionally, it is recommended to measure the heat release rate to understand the fire incident circumstances.

## 6. Conclusions

To test the safety of the previously developed finisher for car park slabs, diverse tests were conducted, such as gas toxicity, heavy metals, chemical resistance and chloride migration tests. From the gas toxicity test outcome, the average elapsed stopping time of lab mice was 14 m 43 s and the SA+PR30% has incombustibility. In the case of the heavy metal evaluation, PAHs, heavy metals, and phthalate plasticisers were not detected, excluding 10 mg/kg of Pb. The developed finisher met all corresponding standards such as KS F 3888-2 and BS EN 71-3. Thus, SA+PR 30% could be implemented as an environmentally friendly finisher for various applications. In terms of the chemical resistance and chloride migration test results, SA+PR30% showed a barrier effect in the chemical environment. Even though there is currently no specific standard for the surface finisher for car park slabs regarding these criteria, these results could be implemented as baseline data when building the standard.

## Figures and Tables

**Table 1:** Mix ratio of developed surface finisher.

Specification	Mix Ratio			
	Waterborne Silicon Acrylic Emulsion	Water	SiO_2_	Polyamide Resin
SA ^(1)^ + PR ^(2)^ 30%	1	0.4	1	0.30

^(1)^ SA, Silicon Acrylic; ^(2)^ PR, Polyamide Resin.

**Table 2:** Heavy metals test acceptance and method.

Evaluation Criteria	Elements (Acceptance)				Test Method
Total PAHs (mg/kg)	(≤10)				KS M 6956
Heavy metals(content) (mg/kg)	Pb		(≤90)		
	Cd		(≤50)		
	Cr^+6^		(≤25)		
	Hg		(≤25)		
Heavy metals(migration) (mg/kg)	Al	(≤70,000)	Cu	(≤7700)	BS EN 71-3
	Sb	(≤560)	Mn	(≤15,000)	
	As	(≤47)	Ni	(≤930)	
	Ba	(≤18,750)	Se	(≤460)	
	B	(≤15,000)	Sr	(≤56,000)	
	Cr	(≤460)	Sn	(≤180,000)	
	Co	(≤130)	Zn	(≤46,000)	
Phthalate plasticisers (%)	DBP		(≤0.1)		KS M 1991
	BBP				
	DEHP				
	DINP				
	DNOP				
	DIDP				

**Table 3:** Gas toxicity test result.

Test No.	Lab Mouse		Mean Elapsed Time (min)
	Gender	Avg. Weight (g)	
No. 1	Female	19	14 min 45 s
No. 2	Female	19	14 min 41 s

**Table 4:** Heavy metals test result.

Evaluation Criteria	Elements (Result)			
Total PAHs (mg/kg)	(-)			
Heavy metals (content) (mg/kg)	Pb		(10)	
	Cd		(-)	
	Cr^+6^		(-)	
	Hg		(-)	
Heavy metals (migration) (mg/kg)	Al	(-)	Cu	(-)
	Sb	(-)	Mn	(-)
	As	(-)	Ni	(-)
	Ba	(-)	Se	(-)
	B	(-)	Sr	(-)
	Cr	(-)	Sn	(-)
	Co	(-)	Zn	(-)
Phthalate plasticisers (%)	DBP		(-)	
	BBP			
	DEHP			
	DINP			
	DNOP			
	DIDP			

**Table 5:** Chloride migration test result.

Mean Value	Plain	SA+PR30%
Applied voltage (U)	25 V	30 V
Anolyte solution (T)	20 °C	20 °C
Specimen thickness (L)	50 mm	52 mm
Penetration depth (x_d_)	14.8 mm	3.3 mm
Test duration (t)	86,400 s	
Non-steady-state migration coefficient	2.2 m^2^/s × 10^−15^	0.4 m^2^/s × 10^−15^
